# Biomarkers of Coagulation Disorders—Where to from Here?

**DOI:** 10.3390/biom16020235

**Published:** 2026-02-03

**Authors:** Benjamin Reardon, Leonardo Pasalic, Emmanuel J. Favaloro

**Affiliations:** 1 Joint Medical Program, School of Medicine and Public Health, University of Newcastle, Callaghan, NSW 2145, Australia; benjamin.reardon@health.nsw.gov.au; 2 Haematology Department, Calvary Mater Hospital Newcastle, Waratah, NSW 2298, Australia; 3 Haematology Department, Institute of Clinical Pathology and Medical Research (ICPMR), NSW Health Pathology, Westmead Hospital, Westmead, NSW 2145, Australia; leonardo.pasalic@health.nsw.gov.au; 4 Sydney Centres for Thrombosis and Haemostasis, Research and Education Network (REN) and ICPMR, Westmead Hospital, Westmead, NSW 2145, Australia; 5 Westmead Clinical School, University of Sydney, Westmead Hospital, Westmead, NSW 2145, Australia; 6 School of Dentistry and Medical Sciences, Faculty of Science and Health, Charles Sturt University, Wagga Wagga, NSW 2650, Australia; 7 School of Medical Sciences, Faculty of Medicine and Health, University of Sydney, Westmead Hospital, Westmead, NSW 2145, Australia

**Keywords:** biomarker, coagulation, bleeding, thrombosis, thrombophilia

## Abstract

Disorders of thrombosis and bleeding contribute to a significant morbidity and mortality burden worldwide. Correctly identifying contributing factors towards either thrombosis or bleeding carries implications for diagnosis, prognosis and management. Although there are established and commonly used biomarkers for both circumstances, the complexity of hemostasis contributes to the wide variability in assay methodology and information provided by each individual assay. There are several emerging biomarkers of thrombosis and hemostasis, which require further evaluation of their roles in diagnosis and management in specific patient populations. This narrative review summarizes established, emerging, and exploratory biomarkers of both thrombosis and bleeding disorders, outlining their biological roles, diagnostic utility, and limitations, with a particular focus on clinical relevance, assay methodology and future directions.

## 1. Introduction

Biomarkers are measurable indicators of biological processes, disease states or responses to therapeutic interventions [1]. Biomarkers of coagulation thus serve as markers of a prothrombotic state or increased bleeding tendency, providing insights into the temporal dynamics of thrombogenesis and potential therapeutic targets [2]. Venous thromboembolism (VTE) and cardiovascular disorders, including acute myocardial infarction and ischemic stroke, are leading contributors to long-term morbidity and mortality with shared common risk factors [3]. The clinical relevance of biomarkers in coagulation is therefore very high.

VTE, including deep vein thrombosis (DVT) and pulmonary embolism (PE), is currently the third leading cause of vascular-related mortality following myocardial infarction and stroke [4,5], where prompt and accurate diagnosis is critical yet challenging due to variable clinical presentations. Combinations of biomarkers of thrombosis, both venous and arterial, may aid in diagnosis, risk stratification and management in the acute care setting. We aim to describe the commonly used biomarkers of bleeding and thrombosis, as well as those that have a growing evidence base but are not widely available or well known.

## 2. Established Biomarkers in Clinical Practice

### 2.1. Standard Coagulation Testing

Blood clotting studies remain a crucial component in the initial assessment of a patient’s coagulation status. Core coagulation evaluation encompasses readily available tests, such as prothrombin time (PT)/international normalized ratio (INR), activated partial thromboplastin time (APTT), fibrinogen, platelet count, D-dimer and sometimes fibrin/fibrinogen degradation products. These tests provide basic but valuable insight into a patient’s risk of bleeding or thrombosis. These tests are not without their substantial limitations. Coagulation is a complex and dynamic process involving multiple pathways, and in vitro testing may not reflect the in vivo processes [6]. Furthermore, the pre-analytical aspects of testing need to be considered for their correct interpretation [7].

### 2.2. APTT and PT/INR

The APTT and PT measure time to clot formation in intrinsic and extrinsic coagulation pathways, respectively [8]. Prolongation of either may be secondary to one or more factor deficiencies, drug effects or the presence of an inhibitor. Normal PT values range from around 9 to 13 s, and prolongation suggests potential reductions in extrinsic and common pathway clotting factors (V, VII, X and prothrombin [II]), anticoagulant effect (such as apixaban, rivaroxaban or warfarin), liver disease or vitamin K deficiency [9]. Systemic inflammation can lead to an overexpression of tissue factors on activated endothelial cells and monocytes, promoting coagulation and thrombin generation [10]. The INR is a mathematical formula that aims to help standardize PT values between laboratories and is primarily used for warfarin (or vitamin K antagonist; VKA) monitoring. The INR is represented by INR = (PT/MNPT)^ISI^, where the PT is the patient’s PT, typically derived from an automated coagulation analyzer, the MNPT is the mean normal PT of at least 20 normal individuals using the same method as the patient’s PT, and the ISI is the international sensitivity index for the PT reagent/instrument in use [11]. The ISI is usually provided by the PT reagent manufacturer.

Normal APTT values range from around 25 to 35 s, and prolongation suggests potential reduction in intrinsic pathway (VIII, IX, XI, XII) and common pathway (II, V, X) clotting factors, the presence of an inhibitor (including lupus anticoagulant), anticoagulant effect (such as apixaban, rivaroxaban and heparin) and possibly liver disease (although the effect may be lower for APTT than for PT, since factor VIII is primarily produced by endothelial cells) [6,9].

Prolongation of both APTT and PT has been described as a biomarker of trauma-induced coagulopathy [12]. Alternatively, shortening of APTT and PT can be seen in some patients; for APTT, this may be due to elevated FVIII levels and is a risk factor for thrombosis [13]. Alternatively, pre-analytical issues may yield both shortening or prolongation of PT and APTT and require exclusion prior to the release of test results.

### 2.3. Fibrinogen

Fibrinogen testing is generally performed as a functional assay based on the time to clot formation following the addition of an excess of thrombin. Fibrinogen is an acute-phase reactant and is elevated in states of inflammation, but is reduced in consumptive coagulopathy and liver disease. Fibrinogen has been reported as a biomarker of disseminated intravascular coagulation (DIC) [14,15], trauma-induced coagulopathy [12] and coagulopathy of sepsis [10]. Hypofibrinogenemia or dysfibrinogenemia may reflect rare congenital disorders of bleeding with a highly heterogeneous phenotype [16]. Alternate acquired deficiencies of fibrinogen are also associated with bleeding [6]. In contrast, elevations in fibrinogen may be associated with thrombosis [2].

### 2.4. Platelet Count

A reduction or increase in platelet counts in patients has many causes [17], and its use as a biomarker of thrombosis or bleeding has shown varying specificity. Severe thrombocytopenia (<10–20,000/µL of blood) has a bleeding risk, which is typically mitigated with platelet transfusion or immunosuppression for immune-mediated causes. Inherited causes of thrombocytopenia should also be considered, but platelet count and blood film assessment are inadequate for a definitive diagnosis. Primary causes of platelet count elevation (>450,000/µL of blood) include myeloproliferative neoplasms and are associated with an increased risk of thrombosis as well as bleeding compared to secondary thrombocytosis [18,19]. Secondary thrombocytosis can be due to iron deficiency, infection, post-splenectomy, inflammatory conditions, malignancy (non-myeloproliferative disorders) or exercise and is rarely associated with thrombotic events, including myocardial infarction or VTE [20].

### 2.5. D-Dimer and Fibrin/Fibrinogen Degradation Products

Fibrin/fibrinogen degradation products (FDPs), including D-dimer, are fragments generated during the enzymatic breakdown of fibrinogen and fibrin by plasmin [21]. D-dimer is a fibrin degradation product derived specifically from clot breakdown, and therefore, is more predictable of thrombosis than other FDPs [22]. Normal D-dimer values are typically less than 500 ng/mL in fibrinogen equivalent units (FEU), but high variability in assay sensitivity and specificity of D-dimer and other FDPs can impact their interpretation [23]. D-dimer is elevated in VTE and DIC [24]. Notably, D-dimer and FDPs in general are not specific to VTE and can be elevated in other hypercoagulable states, including pregnancy, trauma, and malignancy [25,26,27,28].

### 2.6. Thrombophilia Testing

Thrombophilia defines a condition in which there is an increased risk of thrombosis, usually VTE, but may also include arterial thrombosis [29]. Table 1 summarizes the key components of thrombophilia testing, including phenotypic and genetic tests associated with an increased risk of thrombosis. These assays are used as biomarkers of thrombotic disorders. In addition, increases in coagulation factor levels can also reflect thrombosis risk [2].

### 2.7. Markers Normally Associated with Bleeding

Bleeding disorders are highly heterogeneous and vary significantly in bleeding phenotype and severity. Table 2 summarizes the key biomarkers of suspected bleeding disorders. It should be noted that fibrinogen testing is included in this table but has a highly variable clinical phenotype and may be associated with thrombosis [16]. Similarly, while deficiencies in coagulation factors and von Willebrand factor (VWF) are associated with bleeding, high levels of some factors (e.g., FVIII, FIX, VWF) are instead associated with thrombosis [2]. Although vitamin C deficiency and connective disorders have not been listed in the table below, they are underrecognized causes of excessive bleeding, particularly in patients with otherwise unremarkable screening tests of coagulation [41].

## 3. Emerging Biomarkers

### 3.1. Endothelial Activation (Soluble Thrombomodulin)

Thrombomodulin is found on the endothelial surface and plays several roles in hemostasis. Thrombomodulin binds to thrombin, which can then activate protein C and the thrombin-activatable fibrinolysis inhibitor, ultimately attenuating fibrinolysis [51]. Soluble thrombomodulin levels are elevated following injury and have been associated with inferior clinical outcomes in patients following trauma [52,53,54]. An inherited bleeding condition from mutations in the thrombomodulin gene, *THBD*, has been reported to be associated with excessive bleeding following injury, reduced thrombin generation and slower rates of fibrinolysis [55,56,57,58]. Soluble thrombomodulin has also been found to be increased in patients following severe head and neck or extracranial trauma as a biomarker of trauma severity and its ensuing coagulopathy [59].

### 3.2. Thrombin–Antithrombin Complex (TAT)

Thrombin has pleiotropic effects on cellular processes and is canonical in coagulation, converting fibrinogen to fibrin and activating platelets via protease-activated receptors [12,59,60,61]. Thrombin has effects on cellular processes, including vascular permeability, promotion of oxidative stress and upregulation of pro-inflammatory cytokines [62]. Following thrombin formation, the natural anticoagulant antithrombin irreversibly binds to thrombin, leading to thrombin–antithrombin (TAT) complex formation, a validated marker of thrombin generation, particularly in the early stages of coagulation activation [60]. TAT complexes are more easily detected than thrombin [63]. Elevated TAT levels have been associated with coronary slow flow and proposed as a diagnostic tool [64], as well as in patients following myocardial infarction [65]. Elevated TAT levels have also been found in ischemic stroke, with higher levels closely associated with large-volume ischemic stroke, cardioembolic stroke and poor revascularization following stroke, as compared with atherothrombotic or lacunar strokes [63]. Elevated TAT levels have also been found in sepsis-associated coagulopathy [66], following intracranial hemorrhage as a poor prognostic marker, and have higher sensitivity and specificity than D-dimer in detecting VTE post-operatively [67]. TAT detection in the laboratory has some limitations, notably that its short half-life, susceptibility to proteolytic degradation, short time window for detection of elevation following VTE, and notably, some genetic mutations, like those of FVL, may affect TAT levels [68,69,70].

TAT and D-dimer testing has also been studied in patients with hemophilia A and B, following thrombosis after factor VIII or IX administration with marked elevated levels associated with intravascular thrombosis [70,71,72].

### 3.3. Platelet Activation and Adhesion (CD40L, P-Selectin)

Platelets are a key component in thrombosis and thrombotic diseases, where activated platelets mediate thrombus formation and are involved in multiple interactions with vascular cells, inflammatory cytokines, and the coagulation system [73]. Following activation, platelets release over 300 proteins, including P-selectin and CD40L, which are two potential biomarkers of platelet activation.

The CD40 ligand (CD40L) is a transmembrane molecule expressed in T-lymphocytes and platelets [74,75]. Interactions with CD40L are required for many immune responses; CD40L-induced CD40 signaling in B-cells from T-dependent antigens induces BCL6 and Ki67 expression in germinal center B cells, ultimately allowing Ig class switching [74]. The CD40-CD40L system is associated with both prothrombotic and pro-inflammatory effects.

Soluble CD40 is predominantly generated by platelets and contributes to the pathophysiology of atherosclerosis [75,76]. Soluble CD40L enhances platelet activation, aggregation, and platelet–leukocyte conjugation, which contributes towards atherothrombosis [75,76,77,78]. The action and interaction of CD40 and CD40L on endothelial cells is multifaceted and involves upregulation of adhesion molecules and pro-inflammatory cytokines cells [79], whilst also stimulating the expression of tissue factors by monocytes and endothelial cells [80] and contributing to endothelial dysfunction by decreasing nitric oxide (NO) synthesis and augmenting oxidative stress [81]. CD40 is also implicated as a key mediator in platelet-associated transfusion reactions [82,83]. Incubation of platelets with recombinant sCD40L can lead to enhanced P-selectin expression, platelet aggregation, and platelet–leukocyte conjugation [84].

P-selectin is another transmembrane glycoprotein embedded in alpha-granules and stored within Weibel–Palade bodies of vascular endothelial cells [73]. Upon platelet activation, the membrane of alpha-granules merges with the platelet membrane, leading to P-selectin translocation to the platelet surface and eventual cleaving and release of soluble P-selectin [85]. Soluble p-selectin (sP-selectin) is increased in patients with coronary artery disease and shown to be a reliable biomarker across different ethnicities of patients on antiplatelet therapies [86]. sP-selectin is elevated in patients with DVT and PE compared with healthy controls [87,88]. In patients with peripheral vascular disease who require stenting, elevated sP-selectin levels were associated with restenosis, and CD40L was not [89]. Reidl et al. looked at sP-selectin and CD40L, as well as other biomarkers, in cancer patients with VTE, cancer patients without VTE and healthy subjects, and found that sP-selectin was elevated in patients with VTE irrespective of cancer status, which was not found in CD40L and other biomarkers [90].

### 3.4. NETs

Neutrophil extracellular traps (NETs) are associated with the development of thrombosis [91]. As a unique form of cell death resulting in the release of condensed chromatin and granular proteins, NET formation and translocation of reactive oxygen species, myeloperoxidase, histones and neutrophil elastase, they form a scaffold in which platelets, fibrinogen and VWF can adhere and aggregate [92]. Extracellular DNA staining positive for myeloperoxidase, histones and neutrophil elastase has been seen in immunostaining of venous thrombosis [93]. In baboons with iliac vein thrombosis, dotted and diffuse staining of DNA and positive staining of DNA–histone could be seen, which has also been seen in human venous thrombi from surgical samples [92,94]. The role of NETs as a biomarker in venous thromboembolism has been evaluated in a number of studies, including the use of plasma DNA levels, neutrophil elastase and histones (H3Cit-DNA), all of which were elevated in patients with VTE [95,96]. In subsequent studies of the use of plasma DNA and nucleosomes, NET biomarkers were higher in elderly patients with PE compared to those with DVT [97], and subsequently, higher levels of plasma DNA and calprotectin were observed in patients with splanchnic vein thrombosis compared with DVT [98]. NET biomarkers (DNA, myeloperoxidase, nucleosomes) have also been used to predict severity, clinical outcome and hypercoagulability in patients with coronary artery disease and acute myocardial infarction [99,100,101,102,103]. Myeloperoxidase–DNA and histone (H3Cit) have also been shown to be higher in patients with COVID-19 infection compared with healthy controls, with levels correlating with disease severity [104,105]. A number of studies related to the use of NET-based targets are entering the clinical space, particularly in degrading NET formation by use of DNase [106], heparin in its removal of histones in chromatin [92], and JAK1/JAK2 inhibition by abrogating NET formation and reduction in stenosis-induced venous thrombosis [107]. Neutrophil elastase produced by NETs may also be implicated in suppressed fibrinolysis, which may promote persistent microvascular thrombosis and organ dysfunction in critically unwell patients [108].

### 3.5. Extracellular Vesicles

Extracellular vesicles (EVs) are a heterogeneous group of vesicle-derived particles, including phospholipids, tissue factor, P-selectin and other procoagulant proteins, which are released to the extracellular environment in response to inflammatory or proapoptotic factors [109]. EVs are released following a number of stimuli, including IL1α, IL1β, LPS, PMA, TNF α, complement C5b-0, PAI-1, thrombin, staurosporine, ADP, and collagen [110,111,112,113,114]. Many of these stimuli are prothrombotic for EV release; however, they may exert prothrombotic activity themselves [109]. EVs have both direct and indirect effects on platelets, white cells, red cells and coagulation factors [109]. EVs isolated from healthy participants were found to support coagulation in vitro via TF-independent pathways [115]. Measurement of fibrin generation as a surrogate for EV–tissue factor generation has been demonstrated in cancer patients with VTE, with higher fibrin generation being associated with a two-fold increased risk of VTE (HR 2.0, 95% CI 1.1–3.6) [116]. More recently, EVs were associated with elevated interleukin-1B and thrombosis related to anti-phospholipid syndrome [117]. In a prospective study by Antic et al. (2023), 127 patients diagnosed with lymphoma who developed VTE events had higher tissue factor values (224.41 [93.1–446.0] (median [IQR]) vs. 136.43 [109.4–193.4], *p* = 0.036) compared with patients who did not develop VTE, and patients who had aggressive lymphoma had an 8-fold increase in CD11b+/CD16+ neutrophils, a 2-fold increase in CD61+ platelets and a 3-fold increase in CD31+ endothelial cells labeled with TF and extracellular vesicles. Interestingly, P-selectin levels were not found to be different between groups [118].

### 3.6. Plasmin–Alpha2-Antiplasmin Complex (PAP)

Plasmin and α2-antiplasmin (α2-AP) are key regulators of fibrin polymer dissolution into soluble fragments. Free plasmin rapidly combines with α2-AP to form PAP [119]. Elevated PAP levels indicate active thrombolysis and thrombus formation [120,121,122]. PAP more directly reflects plasma plasmin activity and rises earlier than D-dimer in acute VTE [123]. Ma et al. (2025) found that in patients with gynecological malignancy undergoing surgery, PAP area under the receiver operating curve (ROC) was 0.95 preoperatively and 0.941 postoperatively in patients who developed VTE, indicating its use as a predictive biomarker of VTE [123]. However, in another study by Folsom et al. (2003) looking at 308 patients with VTE and 640 controls, there was no significant association of fibrinolytic variables, including PAP, after adjusting for other risk factors and patient age [124].

### 3.7. Epigenetic and Genetic Testing

VTE and arterial thrombotic events are polygenic in nature; however, it is increasingly acknowledged that environmental factors and histone modification contribute towards genomic expression and inheritability [3]. MicroRNAs, long non-coding RNAs and extracellular vesicles have been identified as key elements in regulating gene expression in coagulation [125]. A systematic review by Patsouras and Vlachoyiannopoulos (2019) [126] identified two independent studies examining genome-wide methylation profiles in patients with cardiovascular disease, incorporating a total of 1080 cases, and found that differential DNA methylation in CpG sites in genes is implicated in cardiac function, cardiovascular disease and response to ischemic injury [127,128]. The association of systemic autoimmune disease and cardiovascular disease has been well established [129]. In DNA isolated from patients with rheumatoid arthritis and Kawasaki disease, methylation differences have been seen in pathways involving coagulation and platelet activation, including protein C inhibitor, P-selectin, ICAM1, VWF and MAPK14 genes [130,131].

Epigenetic factors contributing towards thrombosis have also been found in APS, with a significant reduction in methylation in the *IL8* promoter gene and an increased tissue factor compared with healthy controls [126]. Neutrophils isolated from patients with APS and SLE compared with healthy controls have demonstrated 42 differentially methylated CpG sites, which may also contribute to thrombosis [132].

Epigenetics have also been identified as a contributor to cancer-associated thrombosis. Cuff et al. found that hypomethylation of the promoter of the transcription factor of genes associated with coagulation in patients with renal or ovarian clear cell carcinoma was associated with a 2.3-fold increased risk of clinically significant venous thrombosis [133]. Factor X and tissue factor promoter methylation levels were found to be lower in glioma specimens, which correlated with intra-tumor microthrombi and VTE [134,135]. In patients with primary myelofibrosis, methylation of CD18 has been found to be higher than in healthy controls and found to be an independent prognostic factor of thrombosis [136]. This is particularly of note given the interaction of CD11b and CD18 as a leukocyte-specific integrin receptor promoting platelet activation, neutrophil recruitment and endothelial injury [137].

Given the plethora of tests required for the investigation of potential inherited bleeding disorders, genetic testing is being increasingly used to define pathogenic variants and allow for the ultimate classification of disease and its severity [138]. Next-generation sequencing (NGS) allows the ability to test multiple genes of interest (panel-based) and may allow for the discovery of variants previously not known or unclassified (whole exome or whole genome sequencing) [139]. Genetic testing may also assume a key role in bleeding conditions with mild phenotypes that remain unresolved with functional or protein-based testing [140,141]. Genetic testing may also have high clinical utility in the detection of inherited platelet disorders, given that the diagnosis is often more challenging due to high clinical heterogeneity and indistinct laboratory features [142,143,144]. Pezeshkpoor et al. (2022) propose genetic testing as the final definitive test required for precise classification and allowing for greater prediction of clinical bleeding risk [145]. Current trials are underway, investigating the use of early genetic screening in suspected inherited bleeding disorders [146].

### 3.8. Viscoelastic Testing

Viscoelastic testing (VET) is increasingly being used in clinical and research settings to assess hemostasis [6,147]. Whilst there are a number of pre-test clinical scores to assess the risk of hemostasis dysfunction, their reliability and applicability in different patient populations is unclear [148]. A number of studies have used VET to assess hypercoagulability and patients at increased risk of thromboembolism. Maximum clot strength (or maximum amplitude, MA) is the most common component of VET used to assess these conditions. In a retrospective cohort study on hospitalized patients over the age of 65, Zheng et al. (2014) found that the prevalence of venous or arterial thrombotic events was 20.6% in patients with an MA value of >69 mm on VET [149]. In a prospective cohort study on critically ill adult patients, Tartamella et al. (2016) found that the use of a thrombodynamic ratio (MA multiplied by the alpha angle) of >10.6 was associated with a 10.6% rate of VTE [150]. VET parameters have also been shown to reliably detect the hypercoagulable state peripartum and postpartum [151].

Viscoelastic testing is also being researched in its detection of bleeding states, including in patients on direct oral anticoagulants [152], liver disease [153], orthotopic liver transplantation [154], cardiac surgery [155], trauma [156], obstetric-related bleeding [157] and pediatrics [6,147,158]; however, there is a paucity in randomized or prospective studies in some of these populations [147].

### 3.9. Interleukin-6 (IL-6) and Interleukin-7 (IL-7)

The use of additional biomarkers may also be useful in patients with hemophilia experiencing acute bleeding. In a study by Knowles et al. (2023), recent bleeding events within 1 month, as well as acute bleeding presentations, correlated with elevated IL-6 levels in patients with hemophilia A and B, compared with non-bleeding patients with hemophilia [159].

Hemophilia-associated arthropathy is a significant cause of morbidity in patients with limited biomarker testing. In a comparison of rheumatoid- and hemophilia-associated arthropathy, Toenges et al. assessed 42 parameters in 129 male patients, including cytokines, angiogenesis and acute-phase reactants, to better establish a biomarker of arthropathy [160]. When comparing patients with rheumatoid arthritis to patients with hemophilia without associated arthropathy, IL-7 levels were increased non-significantly (*p* = 0.24), and VEGR-1 was decreased (*p* = 0.002); and in hemophilia patients with arthropathy, only serum ferritin was decreased (*p* = 0.008), compared with patients with rheumatoid arthritis.

## 4. Biomarker Validation and Translational Challenges

### 4.1. Regulatory Considerations

Regulators increasingly require a context of use (COU)-anchored framework in the establishment of new biomarkers, allowing for reliability in its specific interpretation and application in a clinical context [161]. Assay development requires testing of precision, accuracy, linearity, selectivity, stability and traceability of biomarkers measured, a large undertaking that requires extensive investment of time and resources [162]. In the development of many of the above biomarkers, testing will also need to include prospective studies and how management (including anticoagulant therapy or factor replacement therapy) affects the assay to avoid verification bias and demonstrate real-world prognostic value [163]. Laboratory capacity constraints may also influence assay uptake and rollout [164].

### 4.2. Point-of-Care Testing

Point-of-care (POC) testing comprises a unique challenge to assay development with specific ISO requirements for implementation [165]. POC testing offers the benefit of rapid turnaround time but is often competing with central laboratory assays which usually have better precision and lower bias [166]. Thus, POC testing head-to-head evaluations with central laboratory testing usually offer acceptable but imperfect agreement due to device-specific reagents, drug effects and calibration affecting universal intervals and clinical cut-offs [167,168].

### 4.3. Integration into Clinical Practice

The process leading to the eventual use of a biomarker in clinical practice can often span decades and can involve international collaboration between laboratories, scientists and clinicians. Vasan (2006) outlines a step-wise approach to biomarker development involving laboratory-based discovery, assay standardization, the establishment of reference intervals among healthy and diseased individuals, validation, and ultimately, regulatory approval for clinical use [2,169].

Multimodal biomarkers, including protein testing, cellular phenotype and genetic testing, may be utilized to better define clinical phenotypes of bleeding or thrombosis [170].

## 5. Conclusions and Future Directions

Biomarkers of coagulation and bleeding provide essential insights into thrombotic and hemorrhagic disorders, spanning established assays as well as comprehensive thrombophilia testing and evaluation of potential bleeding diathesis. Methodological heterogeneity remains a major barrier, where differing reagents, platforms, and interpretive criteria result in variable performance across laboratories. Emerging biomarkers—including soluble thrombomodulin, thrombin–antithrombin complexes, platelet activation markers, NET-derived products, extracellular vesicles, and fibrinolytic markers—offer a more dynamic assessment of coagulation activation and endothelial or inflammatory processes, though analytic variability and assay susceptibility to drug effects require further evaluation.

Future progress requires harmonization of assay methodology, formal establishment of reference intervals, and multicenter validation of emerging biomarkers within clearly defined clinical contexts of use. Integrating biomarker data into structured scoring systems may also strengthen precision when used diagnostically and allow for risk stratification for both thrombosis and bleeding, particularly where multimodal biomarker panels can be implemented. Expanding genetic and epigenetic testing, coupled with biomarker-based phenotyping, will likely refine disease classification and personalized treatment strategies, supporting more robust prediction of thrombotic and bleeding risk in complex or ambiguous cases.

## Figures and Tables

**Table 1:** Summary of biomarkers of thrombophilia.

Test	Abbreviation	Purpose	Type of Assay
**Activated protein C resistance**	APCR	‘Resistance’ to activated protein C inactivation of factors Va and VIIIa. People with APCR are at high risk of VTE.	Several different versions, with some based on aPTT and others based on snake venom activation of coagulation [30,31]. Most cases of APCR are due to genetic causes such as factor V Leiden (FVL). However, depending on the assay, laboratory-detected APCR can also reflect other conditions such as elevated levels of factor VIII and pregnancy-induced hemostasis changes.
**Antithrombin**	AT	The level (‘antigen’) or activity (functional assays) of AT. AT is a serine protease inhibitor, or SERPIN, that operates as a natural anticoagulant to inactivate predominantly thrombin (FIIa) and FXa, and to a lesser extent, the other active enzymes of coagulation biochemistry. Deficiency of AT can lead to thrombophilia, primarily VTE.	AT activity assays are most common and measure the level of functionally active AT through inhibition of FIIa or FXa. Antigen-based assays may be useful to supplement activity assays and help characterize AT defects. *SERPINC1* gene mutation testing: Next-generation sequencing (NGS) or PCR-based testing can be used to distinguish congenital or acquired deficiency.
**Protein C**	PC	The level (‘antigen’) or activity (functional assays) of PC. PC is the vitamin K-dependent zymogen of the serine protease-activated protein C (APC), which serves a critical role in the regulation of thrombin generation by inactivating FVa and FVIIIa, the cofactors for the prothrombinase and tenase complexes, respectively. Deficiency of PC can lead to thrombophilia, primarily VTE.	PC activity assays are most common and measure the level of functionally active PC in chromogenic or clotting assays. Antigen-based assays may be useful to supplement activity assays and help characterize PC defects. *PROC* gene mutation: NGS or PCR-based testing can be used to distinguish congenital or acquired deficiency.
**Protein S**	PS	The level (‘antigen’) or activity (functional assays) of PS. PS is a vitamin K-dependent nonenzymatic cofactor for both APC and tissue factor pathway inhibitor (TFPI), thereby contributing to the downregulation of coagulation in both the initiation and propagation phases. Deficiency of PS can lead to thrombophilia, primarily VTE.	Antigen-based assays for ‘Free’ PS (i.e., not bound to C4b-binding protein (C4BP)) are most commonly performed, as this form of PS reflects the functionally available form of PS. Some centers also evaluate ‘total’ PS levels by antigenic assays. Clot-based PS assays are available but not generally recommended. *PROS1* gene mutation: NGS or PCR-based testing can be used to distinguish congenital or acquired deficiency.
**Factor V Leiden mutation**	FVL	Factor V Leiden mutation represents the most common congenital thrombophilia, affecting 3–8% of the Caucasian population [32]. FVL is a common genetic mutation of FV arising as a single nucleotide polymorphism that abolishes the predominant APC cleavage site in FVa, Arg506 (p. Arg534Gln).	*FV* gene PCR-based assay. Most methods are specific for FVL and may miss less common mutations of the *FV* gene associated with thrombosis. In these circumstances, Sangar sequencing or NGS may be utilized.
**Prothrombin gene mutation**	PGM	The prothrombin gene mutation G20210A occurs in approximately 2–3% of the general population, leading to elevated levels of prothrombin (Factor II), with an increased risk of venous thromboembolism [33].	Genetic testing of *F2* G2021A is typically performed by PCR assays. Most methods are specific for *F2* G20210A and may miss less common *F2* variants associated with thrombosis. In these circumstances, Sangar sequencing or NGS may be utilized.
**Lupus anticoagulant**	LA	Laboratory criteria for antiphospholipid syndrome (APS) associated with venous or arterial thrombosis, or obstetric criteria	At least 2 tests based on different assay principles for phospholipid-dependent prolongation of clotting: activated partial thromboplastin time (APTT) and dilute Russell viper venom time (dRVVT)-based. Of note, there are many factors which can affect the reliability of these assays and thus their interpretation [34].
**anti–beta2-glycoprotein I and anticardiolipin antibodies**	aβ2GPI and aCL	Laboratory criteria for APS associated with venous or arterial thrombosis, or obstetric criteria	Immunological assays and the presence of either immunoglobulin (Ig)G or IgM for aCL and/or aβ2GPI, confirmed after at least 12 weeks.
**JAK2 V617F mutation**	JAK2	*JAK2* V617F constitutes a point mutation localized within the Janus kinase 2. Mutations in *JAK2* lead to cellular proliferation and cytokine release, consistently observed in polycythemia vera and other myeloproliferative neoplasms, as well as increased thrombosis risk secondary to platelet activation, leukocytosis and altered endothelial function [35,36]. Hepatic and splanchnic vein thrombosis have a strong association with *JAK2* mutations [37].	PCR and ddPCR are most commonly used but may miss non-canonical mutations. Sangar sequencing or next-generation sequencing methods may be used for non-*JAK2* V617F mutations.
**Paroxysmal nocturnal hemoglobinuria**	PNH	PNH is characterized by uncontrolled complement activation leading to platelet activation and intravascular hemolysis [38]. Additionally, 29–44% of patients with PNH experience thrombosis, and thrombotic events are associated with high mortality in this population [39].	Flow cytometric-based testing on peripheral blood for detection of loss of GPI-anchored proteins across red cells, monocytes and granulocytes [40]. *PIG-A* mutations are not commonly tested, given the high reliability and availability of flow cytometry-based assays.

Largely adapted from information provided in Favaloro et al. (2025) [29]. Additional material from the table cited references.

**Table 2:** Summary of biomarkers of bleeding disorders.

Test	Abbreviation	Purpose	Type of Assay
**Factor levels**	Factor II, V, VII, VIII, IX, X, XI, XII	Detection of factor deficiency (diagnostic of hemophilia A or B when factor VIII or IX is <40%, respectively) Differentials for low FVIII may include VWD or acquired VWS. Notably, FXII deficiency is not associated with a bleeding disorder, but may explain a prolonged APTT	1-stage clot-based or 2-stage chromogenic assay. Bovine-based chromogenic assays should be used in patients on emicizumab to measure residual factor VIII levels, including recombinant factor VIII [42]. Genetic testing of respective genes by PCR or NGS platforms.
**Inhibitor Detection**	Bethesda/Nijmegen	Detection of factor-specific inhibitor	Usually a clot-based assay with a mix of diluted patient plasma with pooled normal plasma, with Nijmegen modification for comparison of factor-deficient plasma [43]. Chromogenic-based assays used in some centers.
**VWF studies**	VWF:Ag, VWF:RCo, VWF:GPIbM, VWF:GPIbR, VWF:Ab, VWF:CB, VWF:FVIIIB	Detection of quantitative and qualitative VWF	Various assays with the use of assay ratios. Testing performed by ELISA, LIA, CLIA or flow cytometric methods [44].
**VWF Multimer studies**	VWF:Mult	Detection of loss of high- and sometimes intermediate-molecular-weight multimers in type 2A and 2B VWD	Gel electrophoresis.
**Fibrinogen**	Fibrinogen	Detection of hypofibrinogenemia or dysfibrinogenemia	Activity-based assay (Clauss) or antigen-based assay (LIA, EID or ELISA) Genotypes of *FGA*, *FGB* and *FGG* genes [45,46].
**Platelet function analyzer studies**	PFA-100 or PFA-200	Main benefit as a screening test for defects of primary hemostasis, especially VWD. Can detect many abnormalities in platelet function but lacks specificity	High shear rates through an aperture coated with collagen and epinephrine or collagen and ADP (PFA-100 and PFA-200) or prostaglandin E1 (PFA-200) to induce platelet adhesion and activation [47,48].
**Platelet Aggregometry**	LTA, WBA, Multiplate	Most commonly light transmission aggregometry (or whole blood (impedance) aggregometry), used for specificity of platelet defect from specific agonists (e.g., ADP, collagen, ristocetin, adrenaline, arachidonic acid, thrombin, thromboxane A2)	Tests platelet aggregation to particular agonists in vitro with detection of primary and secondary waves, generally using platelet rich plasma (LTA), although whole blood aggregometry (WBA) can be used [49].
**Factor XIII**	FXIII	Detection of FXIII deficiency	Clot solubility, immunological, functional (including ammonia release), or chromogenic assays [50].
**Flow cytometry**	FC	Used to detect the absence of CD41, CD61 and CD42b expression on platelets in Glanzmann thrombasthenia and Bernard Soulier syndrome	Labeling of platelets with antibodies directed against surface glycoproteins.
**Blood Group**	ABO	Detection of type O blood group, which is associated with lower VWF levels and activity	Forward blood group with anti-A, anti-B and anti-A,B.

Abbreviations: LTA, light transmission aggregometry; VWF, von Willebrand factor; VWF:Ab, VWF measured using a monoclonal antibody-based assay; VWF:Ag, VWF antigen; VWF:CB, VWF collagen binding; VWF:FVIIIB, VWF factor VIII binding; VWF:GPIbM, VWF (platelet) recombinant mutated glycoprotein Ib binding; VWF:GPIbR, VWF (platelet) recombinant glycoprotein Ib binding; VWF:RCo, VWF ristocetin cofactor.

## Data Availability

As this manuscript represents a narrative review, or synthesis of existing literature, no new data were created.

## Abbreviations

The following abbreviations are used in this manuscript:

VTE	Venous thromboembolism
PE	Pulmonary embolism
DVT	Deep venous thrombosis
PT	Prothrombin time
APTT	Activated partial thromboplastin time
INR	International normalized ratio
MNPT	Mean normal prothrombin time
DIC	Disseminated intravascular coagulation
FDPs	Fibrinogen degradation products
FEU	Fibrinogen equivalent units
TAT	Thrombin–antithrombin complex
CD40L	CD40 ligand
NET	Neutrophil extracellular trap
EV	Extracellular vesicle
PAP	Plasmin-alpha2-antiplasmin complex
POC	Point of care
NGS	Next-generation sequencing
VET	Viscoelastic testing
IL6	Interleukin-6
IL7	Interleukin-7
LTA	Light transmission aggregometry
VWF	von Willebrand factor
VWF:Ag	VWF antigen
VWF:CB	VWF collagen binding
VWF:FVIIIB	VWF factor VIII binding
VWF:GPIbM	VWF (platelet) recombinant mutated glycoprotein Ib binding
VWF:GPIbR	VWF (platelet) recombinant glycoprotein Ib binding
VWF:RCo	VWF ristocetin cofactor
